# From Volume to Mass: Transforming Volatile Organic Compound Detection with Photoionization Detectors and Machine Learning

**DOI:** 10.3390/s25175314

**Published:** 2025-08-27

**Authors:** Yunfei Cai, Xiang Che, Yusen Duan

**Affiliations:** 1 Shanghai Environment Monitoring Center, Shanghai 200235, China; yfcai@sthj.shanghai.gov.cn (Y.C.); bullet630@163.com (X.C.); 2 NHC Key Laboratory of Health Technology Assessment, Key Laboratory of Public Health Safety of the Ministry of Education, Department of Environmental Health, School of Public Health, Fudan University, Shanghai 200032, China

**Keywords:** random forest, industrial parks, real-time monitoring, mass concentration conversion

## Abstract

(1) Objective: Volatile organic compounds (VOCs) monitoring in industrial parks is crucial for environmental regulation and public health protection. However, current techniques face challenges related to cost and real-time performance. This study aims to develop a dynamic calibration framework for accurate real-time conversion of VOCs volume fractions (nmol mol^−1^) to mass concentrations (μg m^−3^) in industrial environments, addressing the limitations of conventional monitoring methods such as high costs and delayed response times. (2) Methods: By innovatively integrating photoionization detector (PID) with machine learning, we developed a robust conversion model utilizing PID signals, meteorological data, and a random forest’s (RF) algorithm. The system’s performance was rigorously evaluated against standard gas chromatography-flame ionization detectors (GC-FID) measurements. (3) Results: The proposed framework demonstrated superior performance, achieving a coefficient of determination (R^2^) of 0.81, root mean squared error (RMSE) of 48.23 μg m^−3^, symmetric mean absolute percentage error (SMAPE) of 62.47%, and a normalized RMSE (RMSE_norm_) of 2.07%, outperforming conventional methods. This framework not only achieved minute-level response times but also reduced costs to just 10% of those associated with GC-FID methods. Additionally, the model exhibited strong cross-site robustness with R^2^ values ranging from 0.68 to 0.69, although its accuracy was somewhat reduced for high-concentration samples (>1500 μg m^−3^), where the mean absolute percentage error (MAPE) was 17.8%. The inclusion of SMAPE and RMSE_norm_ provides a more nuanced understanding of the model’s performance, particularly in the context of skewed or heteroscedastic data distributions, thereby offering a more comprehensive assessment of the framework’s effectiveness. (4) Conclusions: The framework’s innovative combination of PID’s real-time capability and RF’s nonlinear modeling achieves accurate mass concentration conversion (R^2^ = 0.81) while maintaining a 95% faster response and 90% cost reduction compared to GC-FID systems. Compared with traditional single-coefficient PID calibration, this framework significantly improves accuracy and adaptability under dynamic industrial conditions. Future work will apply transfer learning to improve high-concentration detection for pollution tracing and environmental governance in industrial parks.

## 1. Introduction

Volatile organic compounds (VOCs) are a class of organic compounds with high vapor pressure at ambient temperatures, making them prone to volatilization into the atmosphere. Industrial parks, characterized by concentrated emissions from industrial activities, such as waste gas discharge and solvent volatilization, are significant VOC sources. As precursors to ozone and secondary organic aerosols (SOAs) [1,2,3,4], VOCs contribute to regional air pollution through photochemical reactions, significantly affecting local air quality [5,6]. Furthermore, reports from the World Health Organization (WHO) and the International Agency for Research on Cancer (IARC) [7,8], long-term exposure to specific VOCs such as benzene and formaldehyde can increase the risk of disease, including leukemia and nasopharyngeal cancer. Precise monitoring of VOCs emissions in industrial parks is essential not only for pollution source tracing and liability determination, but also for health risk assessment and targeted environmental governance [9,10,11,12], playing a vital role in improving air quality and safeguarding public health. According to China’s *2024 Ecological Environment Statistical Report* [13], industrial-source VOCs emissions account for 32.8% of the total anthropogenic emissions, which highlights the urgent need to develop effective monitoring technologies for industrial parks.

Currently, online VOC monitoring in Chinese industrial parks primarily relies on gas chromatography–mass spectrometry (GC-MS) or gas chromatography–flame ionization detectors (GC-FID). These devices are known for their high sensitivity, wide linear range, and stability [14], particularly in analyzing individual VOC species [15,16]. However, they also have several drawbacks: Firstly, the equipment is costly, with GC-MS and GC-FID systems typically priced in the hundreds of thousands of dollars, representing a significant initial investment for many industrial parks and monitoring agencies. Additionally, maintenance costs are substantial, requiring regular replacement of consumables (e.g., chromatography columns, carrier gases) and professional maintenance, further increasing operational expenses. Secondly, these devices lack temporal representativeness, with each analysis taking between 20 min and 1 h, making them inadequate for real-time monitoring and rapid response. Moreover, they have stringent environmental requirements, making large-scale deployment within industrial parks challenging. Lastly, they require highly skilled operators who have undergone specialized training, which limits their application in grid-based monitoring.

Instruments equipped with a photoionization detector (PID) have gradually become an important tool for the rapid screening of VOCs due to their millisecond response times and low costs [17,18,19,20]. These instruments ionize VOC molecules using ultraviolet lamps and can quickly output volume concentrations (in μmol mol^−1^/nmol mol^−1^). They can also monitor individual gases through conversion coefficients, significantly compensating for the lack of timeliness in traditional technologies. However, PID technology has two inherent flaws: ① The differences in ionization energies (IP) of various species in mixed VOCs make it difficult to accurately characterize the dynamic response of multi-component systems through measurement results. ② Since environmental regulatory requirements in China typically use mass concentration (mg m^−3^) as the benchmark, and the composition of VOCs in industrial parks is complex and dynamically changing (e.g., changes in wind direction can lead to variations in pollutants), traditional single-calibration coefficients cannot adapt to the synergistic changes in multiple components and the interference of environmental parameters (such as seasonal fluctuations in temperature and humidity), resulting in significant deviations between PID output data and regulatory standards.

In recent years, the rapid development of machine learning technologies [21,22,23,24] has attracted increasing attention in the field of environmental monitoring, offering innovative solutions to the challenges mentioned above. Machine learning algorithms can automatically learn and identify complex patterns and relationships in data, enabling the precise prediction and classification of unknown samples. For instance, machine learning has been applied to optimize sensor array design [25], classify VOCs [26], and monitor soil VOC fluxes in real-time using PID [27]. Among various algorithms, Random Forest (RF) demonstrates unique advantages: (1) Its ensemble learning architecture can effectively mitigate overfitting caused by high variability in environmental data. (2) The inherent nonlinear modeling capability aligns well with the complex interactions between PID signals and meteorological parameters. (3) Feature importance analysis can provide interpretable insights into key influencing factors. These characteristics make RF particularly suitable for developing robust calibration models that can adapt to heterogeneous conditions in industrial monitoring.

This study innovatively integrates PID technology with machine learning to address critical scientific challenges in real-time VOCs monitoring within industrial parks. The research focuses on the following: (1) constructing a dynamic calibration model to overcome the technical bottleneck of converting PID signals to mass concentrations; (2) systematically comparing the performance of five algorithms—support vector regression (SVR), polynomial regression (PR), decision tree (DT), gradient-boosting decision tree (GBDT), and RF—with an emphasis on evaluating key metrics such as adaptability to coupled multi-environmental parameters, nonlinear feature extraction capability, and model interpretability; and (3) establishing an intelligent monitoring system through multi-region field validation that integrates real-time performance (minute-level response), cost-effectiveness (over 80% cost reduction), and high accuracy (R^2^ > 0.8). This technical solution will provide next-generation technological support for pollution source tracing, dynamic health risk early warning, and precise environmental governance in industrial parks, holding significant practical implications for advancing the intelligent transformation of environmental regulations.

The developed dynamic calibration framework not only enables the accurate conversion of PID data to mass concentrations, but also supports real-time monitoring and alarm functions. Calibrated PID data can be used to continuously monitor VOC concentrations and trigger alarms when levels exceed a predefined threshold (e.g., 1000 μg m^−3^). This capability is essential for the timely detection and management of pollution events, ensuring environmental compliance and protecting public health. The system’s rapid response and cost-effectiveness make it a valuable tool for industrial parks aiming to enhance environmental governance and reduce operational costs.

## 2. Materials and Methods

### 2.1. Study Sites and Equipment

This study selected three representative industrial parks as monitoring sites (A, B, and C) based on their distinct industrial profiles and VOCs emission characteristics:

Site A

• Located in Shanghai, China

• Represents a typical chemical industry cluster with dense manufacturing facilities

• Characterized by high-intensity VOC emissions, primarily from pharmaceutical and coating production processes

Site B

• Situated in a mixed industrial-residential area

• Characterized by water treatment plants and new material manufacturing facilities

• Exhibits moderate VOC emission levels with significant diurnal variations

Site C

• Dominated by mechanical processing industries

• Exhibits relatively stable, yet compositionally complex, VOC emissions

• Reflecting contributions from both industrial activities and urban background sources

The equipment configuration included the following:

(1) The PID online monitoring systems included eight devices from four manufacturers (Brands Y, Z, A, and S), each equipped with 10.6 eV UV lamps and dehumidification components [28]. PID technical specifications: detection range: 0–20 μmol mol^−1^ (isobutylene equivalent); response time: <3 s; humidity compensation: from 0 to 95% RH; zero drift: ±1% F.S./24 h.

(2) The reference GC-FID system was utilized for standardized VOC quantification, with alarm thresholds set at 1000 μg m^−3^ based on the sum of individual compound concentrations.

### 2.2. Experimental Design

#### 2.2.1. Equipment Installation, Calibration, and Comparison

During the comparison test, both the PID and GC-FID systems operated simultaneously. The correlation between VOC concentrations measured using the PID and GC-FID systems was assessed by calculating the coefficient of determination (R^2^). To ensure data validity, only measurements with a validity exceeding 80% were included. In line with relevant maintenance regulations, the GC-FID system was calibrated weekly using standard gases to maintain stable operation. Data were reviewed daily to guarantee their integrity and reliability. The flow rate of the PID device was calibrated monthly throughout the monitoring period. Additionally, the PID system can switch to a 15 min rolling average output at the software level, offering flexibility to meet both regulatory requirements and real-time monitoring needs. During GC-FID maintenance periods (weekly calibration and unscheduled repairs), PID data were continuously recorded, but were excluded from model training and validation to preserve the reliability of reference data. The PID system can provide 15 min rolling averages for real-time monitoring while maintaining hourly averages for regulatory compliance.

#### 2.2.2. Algorithm Optimization

All models were implemented using the Python programming language (version 3.9). The dataset was collected over a 15-month period from December 2021 to May 2023, comprising 3532 valid samples across three sites. To ensure a representative concentration distribution, the data into 60% for the training set, 20% for the validation set, and 20% for the test set using stratified sampling. For model development, we employed Python 3.8 with scikit-learn 1.0.2 was employed, utilizing GridSearchCV for hyperparameter optimization with 10-fold cross-validation. The input features included the volume fraction data from the PID devices, as well as wind direction, wind speed, temperature, humidity, and atmospheric pressure, were used as input features, while the mass concentration data from the GC-FID system were used as the target variable. GridSearchCV was employed to optimize the hyperparameters of the five models mentioned above, balancing model complexity (e.g., tree depth, learning rate) to avoid overfitting or underfitting, thereby enhancing prediction performance and stability. The specific settings are shown in Table 1.

To better understand the contribution of each input feature to the model’s prediction, feature importance analysis was conducted for all models. The correlation coefficient between each feature and the target variable (VOC mass concentration) was calculated to assess its influence on the model’s predictions. This helps clarify the impact of meteorological parameters on VOC concentration prediction.

#### 2.2.3. Data Flow and Pseudocode

**Figure 1:**
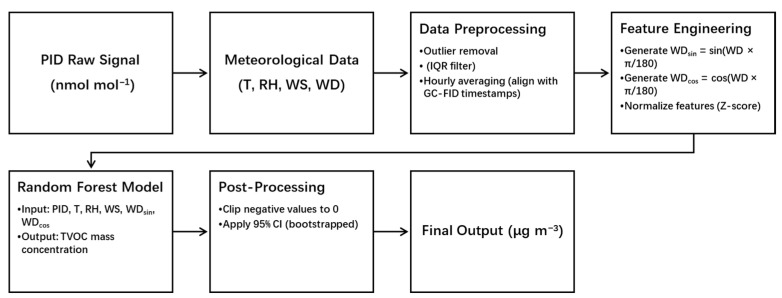
Data flow pipeline from raw PID signals (nmol mol^−1^) and meteorological inputs to final VOC mass concentrations (μg m^−3^).

To clarify the data processing pipeline, Figure 1 presents a block diagram illustrating the workflow from raw sensor input to the final VOC mass concentration output.

#### 2.2.4. Computational Resources

The configuration of the existing equipment is as follows: CPU: Cortex-A8; Memory: 512 MB; Storage: 4 GB; Operating System: Linux.

Our research aims to enhance existing PID equipment rather than replace it entirely with new hardware. This approach not only reduces costs, but also facilitates rapid deployment and implementation. The model operates efficiently on resource-constrained embedded systems, making it well-suited for real-time monitoring applications. Additionally, the use of TensorFlow Lite optimizes model deployment on these systems, ensuring low-latency inference without requiring a GPU.

### 2.3. Data Analysis

#### 2.3.1. Data Preprocessing

We cleaned the collected data to remove outliers and missing values. Next, we converted the volume fraction data measured by the PID device to match the time resolution of the GC-FID system by calculating hourly averages for comparative analysis. For the total concentration values obtained from the GC-FID system, the following formula was used to convert the monitoring concentrations:

$$C_{fid}=\sum_{i=1}^{n}\frac{C_{i}}{{CF}_{i}}$$

Here, $C_{i}$ represents the volume fraction (nmol mol^−1^) of VOCs that the PID device can detect (used in correlation analysis) or the mass concentration (μg m^−3^) of individual VOCs (used for model building and validation), and ${CF}_{i}$ is the calibration coefficient of the compound on the PID sensor [29]. Compared to the simple summation of individual single factors, this method significantly improves the correlation between the sensor and the GC-FID system.

Due to the inherent limitations of the GC-FID system’s measurement principle, the monitoring analysis is not continuous. The GC-FID system used in this study operates on a 30 min analysis cycle during the last half of each hour. During this 30 min period, the device performs eight sampling events, each lasting two minutes, totaling approximately 16 min of actual sampling.

The data obtained from this analysis are referred to as hourly averages. The hourly averages of the PID device are continuous, aggregated from minute-level or second-level data. In this study, the minute-level PID data corresponding to the GC-FID sampling periods were selected to generate new hourly values, which were then compared with the hourly averages from the GC-FID system.

To comply with China’s environmental regulatory standards, this study used hourly averages. These hourly averages from the PID device are continuous and aggregated from minute- or second-level data. Specifically, minute-level PID data corresponding to the GC-FID sampling periods were selected to generate new hourly values, which were then compared with the hourly averages obtained using the GC-FID system.

#### 2.3.2. Correlation Analysis

The correlation between VOC concentrations measured by the PID and GC-FID was evaluated by calculating the coefficient of determination R^2^ to evaluate their linear relationship. Additionally, the correlation between meteorological parameters (wind direction, wind speed, temperature, humidity, and atmospheric pressure) and VOC concentrations were analyzed to determine the impact of these factors on the monitoring data.

#### 2.3.3. Model Building and Validation

The dataset was divided into training and testing sets at a ratio of 8:2. Model stability was evaluated using 10-fold cross-validation on the training set. Model performance was assessed by calculating the mean squared error (MSE), root mean squared error (RMSE), and coefficient of determination (R^2^). To further enhance the robustness of our evaluation, we calculated the symmetric mean absolute percentage error (SMAPE) and the normalized root mean squared error (RMSE_norm_), which provide additional insights into model performance and are less sensitive to data scale. Additionally, to estimate the prediction uncertainty, we employed bootstrapping methods to derive 95% confidence intervals (CI) for the RMSE. This comprehensive approach to evaluating model performance and uncertainty ensures that our findings are reliable and generalizable across different environmental conditions, as further validated at Sites B and C.

## 3. Results

### 3.1. Comparison of PID and GC-FID System and the Impact of Meteorological Factors

#### 3.1.1. Comparison of PID and GC-FID Systems

In the comparative test conducted at Site A, the volume fractions (nmol mol^−1^) of VOCs obtained from the PID devices showed a significant correlation with those measured by the GC-FID system. The highest correlation coefficient was observed for Device Z-1 (R^2^ = 0.92). However, notable performance differences were evident among PID devices from different manufacturers (Table 2). For instance, Device Y-2 exhibited a slope (0.94) close to the ideal value, while Device Z-1 exhibited an anomalously high slope (6.07) due to calibration deviation, indicating systematic differences in signal response between devices. The RMSE values for GC-FID and PID data ranged from 18.7 to 59.6 nmol mol^−1^, confirming the effectiveness of PID in capturing emission trends, despite its inherent technical limitations of not being able to directly output mass concentration.

#### 3.1.2. Impact of Meteorological Parameters on VOCs Concentrations

**Figure 2:**
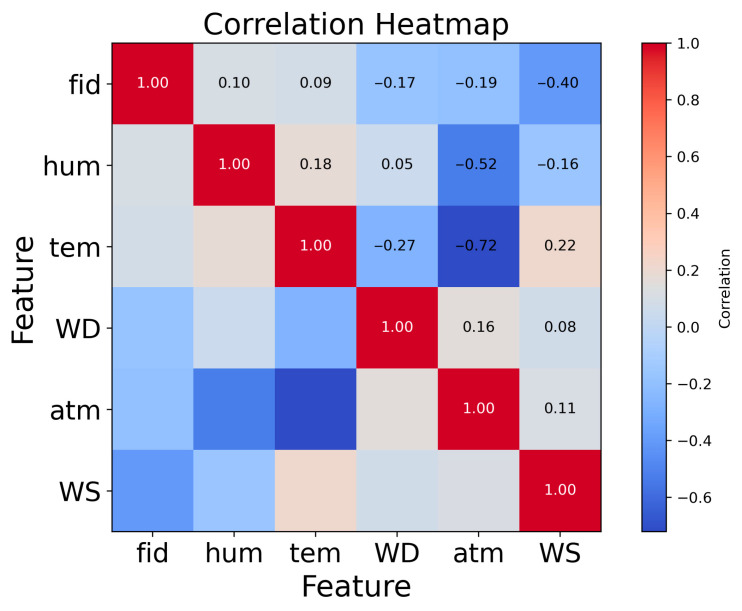
Correlation heatmap between VOC concentrations (GC-FID) and meteorological parameters (temperature, humidity, wind direction). Red and blue indicate positive and negative Pearson correlation coefficients, respectively.

Meteorological parameters significantly influence the monitoring data of VOC concentrations. Figure 2 illustrates that, during the comparison process, the negative correlation (−0.1748) between the GC-FID system concentration and wind direction indicates that changes in wind direction substantially impact VOC concentrations. This negative correlation reflects the combined effects of the pollution source location at Site A, variations in wind speed, topographical features, and regional transport phenomena. An increase in temperature generally enhances the volatility of VOCs, while humidity can affect the detection sensitivity of PID devices. Although the PID device is equipped with dehumidification components, its detection signals may still be somewhat interfered with under high-humidity conditions. By integrating temperature and humidity data, the model can effectively correct these interferences and improve prediction accuracy.

### 3.2. Comparison of Model Performance and Analysis of the Impact of Meteorological Factors

#### 3.2.1. Comparison of Model Performance Comparison

In this study, a systematic evaluation was conducted on five machine learning algorithms (Table 3). The results reveal that the RF model demonstrated the best performance in converting volume fraction to mass concentration, achieving a coefficient of determination (R^2^) of 0.81, a root mean square error (RMSE) of 48.23 μg m^−3^, a symmetric mean absolute percentage error (SMAPE) of 62.47%, and a normalized RMSE (RMSE_norm_) of 2.07 × 10^−2^ on the test sets, significantly outperforming the other models. To provide a more robust estimation of the model’s predictive performance, we also calculated the 95% confidence intervals for the RMSE using bootstrapping methods, which ranged from 28.94 μg m^−3^ to 55.19 μg m^−3^. This indicates that 95% of the predictions are expected to fall within this range, adding a measure of reliability to our results. The SMAPE and RMSEnorm metrics offer more stable insights compared to the mean absolute percentage error (MAPE), especially for skewed or heteroscedastic data, as they are less sensitive to low values and normalize the error relative to the data range.

The high MAPE (129.95%) reflects the logarithmic concentration distribution: for low-concentration samples (<50 μg m^−3^), an absolute error of 10 μg m^−3^ results in a relative error exceeding 20%. When samples with concentrations below 100 μg m^−3^ are excluded, the MAPE decreases to 35.2%, suggesting that MAPE may be better suited for heterogeneous datasets. To provide a more comprehensive evaluation of model performance, especially in the presence of skewed or heteroscedastic data, we also introduced SMAPE and the RMSE_norm_. The SMAPE, which is less sensitive to low values and provides a more balanced measure of error, was calculated to be 62.47%. The RMSE_norm_, which normalizes the RMSE by data range, was found to be 2.07 × 10^−2^. These metrics offer a more stable and nuanced understanding of the model’s performance.

The RF algorithm has two core strengths:

(1) Anti-overfitting capability: This method integrates multiple decision trees and employs voting to reduce noise interference, resulting in less than a 5% difference in R^2^ between the training and test sets.

(2) Nonlinear modeling capability: It adaptively captures the complex interactions among PID signals, temperature, and humidity (e.g., higher temperatures accelerate VOC volatility, while higher humidity suppresses PID ionization efficiency).

In comparison, the other models exhibit significant limitations: SVR, despite its multivariate adaptability, is restricted by the use of linear kernel function (R^2^ = 0.79; MAPE = 221.43%; RMSE_norm_ = 4.23 × 10^−2^), which hampers its ability to fit the nonlinear characteristics **inherent** in environmental data. PR can model nonlinear relationships through higher-order terms (R^2^ = 0.72; RMSE = 58.70 μg m^−3^; RMSE_norm_ = 2.29 × 10^−2^), but it is prone to overfitting, resulting in poor generalizability (RMSE = 58.70 μg m^−3^). DT, although highly interpretable with visualizable feature importance, lacks model complexity (R^2^ = 0.65) and is unable to handle the coupling effects of multiple variables. GBDT’s residual iterative optimization (R^2^ = 0.80; RMSE_norm_ = 2.08 × 10^−2^), but still underperform compared to RF in fitting high-concentration samples.

By incorporating SMAPE and RMSE_norm_ into our evaluation framework, we provide a more stable and comprehensive assessment of model performance, which is crucial for regulatory and decision-making purposes.

**Figure 3:**
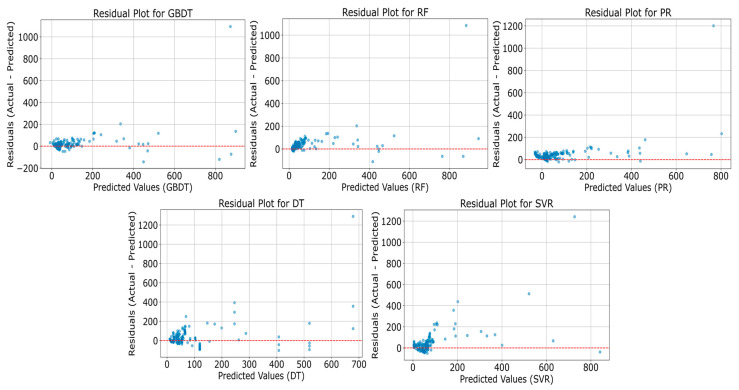
Comparison of model performance using residual plots. This figure displays residual plots for five machine learning models: GBDT, RF, PR, DT, and SVR. Each subplot illustrates the distribution of residuals between the predicted values and the actual TVOC concentrations. The blue dots represent the residuals for each prediction made by the model, which are the differences between the actual TVOC concentrations and the predicted values. The red line represents the line where the residuals equal zero, indicating perfect predictions where the actual values match the predicted values. The closer the blue dots are to this line, the better the model’s performance.

**Figure 4:**
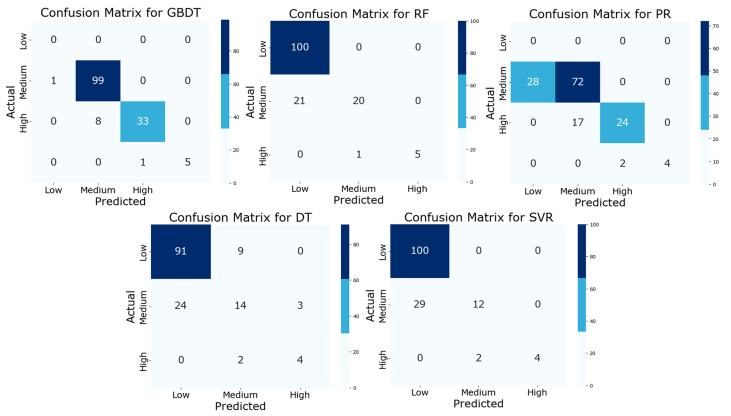
Comparison of model performance using confusion matrices. This figure presents the classification accuracy of five machine learning algorithms for VOC monitoring through confusion matrices. The algorithms include GBDT, RF, PR, DT, and SVR, with the goal of evaluating their predictive performance across various concentration levels.

In our study, we constructed residual plots (Figure 3) and confusion matrices (Figure 4) to evaluate the performance of various machine learning models. The residual plots assess predictive accuracy by comparing the differences between the predicted values and actual measurements of VOC mass concentrations. The confusion matrices illustrate how each model classifies VOCs into low-, medium-, and high-concentration categories, thereby evaluating their predictive capabilities across different concentration levels. Figure 3 displays the residual plots for five machine learning models, including GBT, RF, SVR, PR, and DT. These plots assess the predictive performance of the models by comparing the differences between the predicted values and the actual measurements of VOC mass concentrations.

The plots reveal that the GBT, RF, and SVR models have residuals that are more randomly distributed around the zero line, with most residuals clustering near this line. This pattern indicates that these models exhibit relatively good accuracy in predicting VOC concentrations. However, the residual plots for the PR and DT models display some systematic biases, particularly at the higher end of the predicted values, suggesting that these models may have limitations when predicting high VOC concentration levels.

The residual plot for the DT model, in particular, displays a distinct funnel shape, suggesting that the model produces larger errors when predicting lower concentrations and smaller errors when predicting higher concentrations. Meanwhile, the residual plot for the PR model reveals a trend of increasing residuals as predicted values rise, potentially indicating instability in the model when handling high-concentration data.

In our study, we created confusion matrices (see Figure 4) to illustrate how different machine learning models convert VOC volume fractions into mass concentrations. These matrices provide a clear depiction of how each model classifies VOCs into low-, medium-, and high-concentration categories.

The confusion matrices in Figure 4 demonstrate that the RF model performs best. It achieves the highest accuracy and the lowest error rate across all concentration levels. This aligns with our earlier findings, where the RF model outperformed others in key metrics such as R^2^, RMSE, and SMAPE.

These matrices also reveal the strengths and weaknesses of each model. For example, the DT and GBDT models struggle with medium concentration levels, possibly due to difficulties in handling complex relationships. The SVR and PR models underperform compared to the RF model across all categories, further highlighting the RF model’s effectiveness in managing varied emissions in industrial settings.

#### 3.2.2. Analysis of the Impact of Meteorological Factors

**Figure 5:**
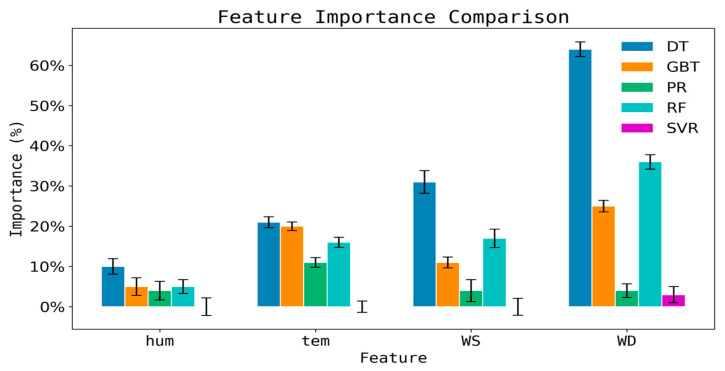
Assessment of the importance of meteorological factors by various models.

In this study, the feature importance of five machine learning algorithms was analyzed. As shown in Figure 5, there are significant differences in the impact of meteorological factors across different models. In the RF model, WD and WS are the most important features, with WD being more influential than WS. This indicates that RF can effectively utilize WD information to optimize the prediction of diffusion paths. The DT model is most sensitive to WD, with its importance ranking higher than temperature and WS, reflecting the high sensitivity of the single-tree structure to changes in WD. In the GBDT model, the importance of WD and temperature is comparable and higher than that of WS, suggesting that GBDT suppresses noise interference to some extent through residual optimization. In contrast, SVR and PR models exhibit lower sensitivity to meteorological factors, with generally low importance values for each feature. This may be due to the limitations of linear or low-order kernel functions in capturing nonlinear relationships.

Overall, the RF model demonstrates significant advantages in handling complex environmental data, particularly in terms of multivariate adaptability and nonlinear fitting capabilities. Future research can further explore multi-sensor fusion and dynamic calibration mechanisms to enhance model performance and generalizability. Additionally, understanding the differences in model sensitivity to meteorological factors is crucial for optimizing environmental monitoring models and improving prediction accuracy.

### 3.3. Case Analysis of High-Concentration Pollution Events

**Figure 6:**
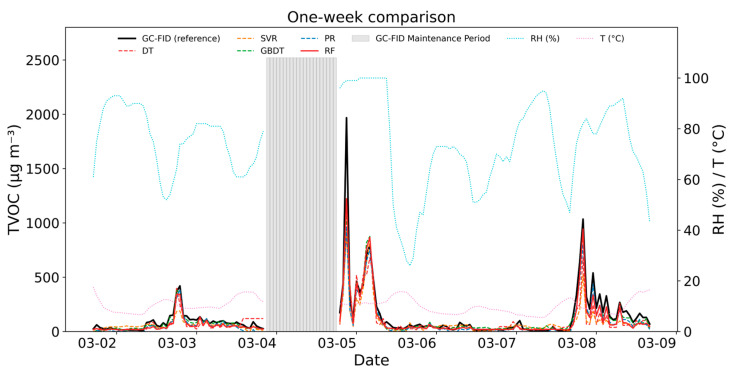
One-week comparison of VOC concentrations (μg m^−3^) measured by GC-FID (reference) versus PID calibrated with RF, GBDT, DT, PR, and SVR (2–8 March 2022). The RF (red line) shows the closest alignment with GC-FID, particularly during high-humidity daytime conditions. Data gaps (approximately 30%) correspond to scheduled maintenance periods of the GC-FID instrument, during which no reference data were available.

To enable readers to quickly evaluate real-world model performance, Figure 6 presents a continuous one-week comparison (2–8 March 2022) of VOC concentrations measured using GC-FID and PID sensors calibrated using five different algorithms. Visual inspection reveals that the RF model (red line) stays within this band more consistently than the other algorithms, while also accurately capturing sharp emission peaks with the smallest deviation.

To further validate the effectiveness of the RF model in practical applications, we conducted a detailed analysis of a specific pollution event shown in Figure 6. This event took place around 9:00 PM on 4 March 2022, when the PID device detected a sudden spike in VOC volume fraction, reaching 869.76 nmol mol^−1^. The model converted the PID measurements into a mass concentration of 1223.4 μgm^−3^ and compared these values to the actual measurements from the GC-FID system.

The pollution event occurred near a chemical plant at Site A, with primary emissions comprising aromatic hydrocarbons and alkanes. During the event, meteorological conditions included a temperature of 6.9 °C, 74% humidity, wind speed of 2.2 m s^−1^, and wind direction of 83.1°. These factors contributed to the local accumulation of VOCs, causing a sudden spike in their concentration. The GC-FID recorded a peak concentration of 1967.68 μg m^−3^, with 1,3-butadiene as the dominant compound (787.3 μg m^−3^, representing 40% of the total concentration), as shown in Table 4. This unusually high concentration of 1,3-butadiene was not observed in the hours before or after the event, which were mainly characterized by typical species such as toluene, xylene, and isopentane. The rarity of such elevated 1,3-butadiene levels in the training data resulted in the less accurate calibration of the RF model for this compound.

### 3.4. Model Generalizability and Cross-Scenario Challenges

#### 3.4.1. Cross-Site and Cross-Seasonal Validation

**Figure 7:**
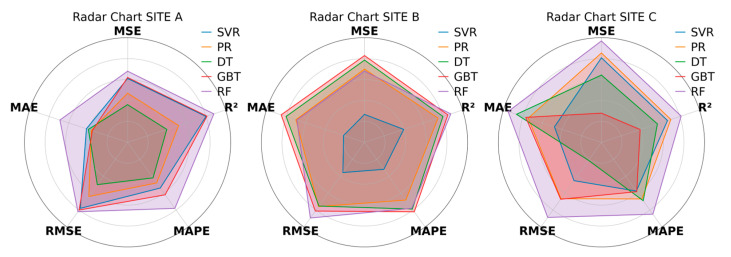
Comparison of indicators for different models.

To comprehensively assess the model’s generalizability, this study collected data from Sites B and C (from January to May 2023). The data from Site A during the winter of 2022 (from December 2021 to March 2022) were used as a basis to verify the model’s adaptability to different seasons and emission characteristics. After normalizing the evaluation indicators for the three sites, the radar chart (Figure 7) clearly shows that the RF model outperformed all other models, achieving the highest scores in most evaluation metrics, especially MSE, RMSE, MAE, and R^2^, while maintaining a relatively low MAPE. GBDT also demonstrated strong performance, with high scores in most metrics, although its MAPE was relatively high. In contrast, SVR consistently performed poorly across all charts and indicators, with generally low scores. PR and DT showed mediocre performance, with PR outperforming SVR, but still lagging behind DT, GBDT, and RF. Overall, the radar chart clearly indicates that RF was the best-performing model, followed by GBDT, while SVR consistently underperformed. Given RF’s superior performance, it was used uniformly for modeling and analysis at all three sites.

#### 3.4.2. Generalizability Analysis—Pollutant Concentration

According to the investigation, the emission sources at Site A are primarily concentrated in pharmaceuticals, coatings, and fine chemicals, which have high VOC emissions and relatively concentrated sources. In contrast, Site B’s emission sources include water treatment, chemical manufacturing, and new materials, while Site C’s sources are dominated by mechanical processing and residential activities. Sites B and C are located farther from the emission sources. These differences result in variations in VOC concentrations. Analysis of the RF models at the three sites (Table 5) shows that Site A exhibits higher concentrations with extreme values, where the model performs well, yielding a higher R^2^ value. However, this may obscure prediction errors at low-value points, leading to a higher RMSE. Additionally, monitoring at Site A was conducted in winter, whereas monitoring at Sites B and C occurred in summer; this discrepancy may be related to temperature-induced baseline drift of the PID [17]. Future iterations should incorporate seasonal calibration cycles. In comparison, Sites B and C have lower concentrations, with data closer to the lower limit of the model’s prediction range, resulting in larger relative errors and lower R^2^ values. Nevertheless, these sites have lower RMSE values, indicating smaller absolute prediction errors.

**Figure 8:**
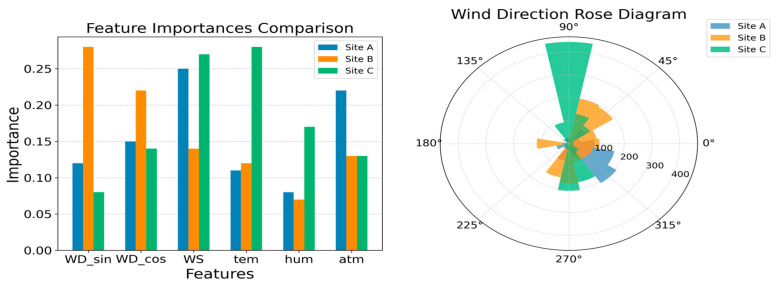
Comparison of feature importance and wind rose diagram.

To investigate the model’s dependence on input features, this study analyzed temperature, humidity, wind direction, and wind speed to assess their impacts on prediction accuracy (Figure 8).

Figure 8 (left) compares the feature importance across the three sites (A, B, and C). WDsin and WDcos represent the sine and cosine components of wind direction, respectively. These features are used because they effectively capture the periodicity and directionality of wind direction by converting angular data into a numerical format that is easier for the model to process. This approach not only resolves the periodic boundary issue inherent in angular data, but also enhances the numerical stability and interpretability of the model, thereby improving the quantification of wind direction’s impact. Consequently, the model can more accurately understand and predict the influence of wind direction on target variables, such as VOC diffusion. The right panel displays a wind rose diagram, illustrating the frequency distribution of wind direction at each site. The analysis reveals that wind direction features (WDsin and WDcos) have the greatest impact on the target variable at Site B, where the dominant wind direction is easterly (90°), confirming the critical role of wind direction in VOC diffusion. In contrast, at Sites A and C, the dominant wind direction is westerly (270°), but the wind speed (WS) feature contributes more significantly to the target variable at these locations.

In summary, the generalizability of the RF model is limited by two factors: (1) differences in regional emission characteristics, as the PID response characteristics of various VOC components have not been fully modeled; and (2) the coupling effects of meteorological conditions, with easterly winds (90°) at Site B promoting pollutant dispersion, while westerly winds (270°) at Site A lead to local accumulation. Future work could involve transfer learning to extract common features across regions or incorporating emission source types as categorical variables to improve prediction accuracy in heterogeneous scenarios.

## 4. Discussion

The present study demonstrates that integrating a low-cost PID with a RF-based dynamic calibration framework can reliably convert VOC volume fractions (nmol mol^−1^) to mass concentrations (μg m^−3^) with a response time on the order of minutes and at approximately one-tenth the cost of conventional GC-FID systems. The RF model achieved the best overall performance (R^2^ = 0.81; RMSE = 48.23 μg m^−3^), outperforming four alternative algorithms, and maintained robustness across three industrial parks with heterogeneous emission profiles (R^2^ = 0.68–0.69). Two mechanistic insights emerged: First, the RF model’s ensemble structure effectively suppressed PID signal noise caused by variable ionization potentials of multi-component VOC mixtures, a known limitation of single-coefficient calibration [17]. Second, the nonlinear interactions between meteorological variables (wind direction, wind speed, temperature, and humidity) and PID response were successfully captured, confirming that environmental covariates are indispensable for accurate mass conversion in dynamic industrial settings.

Our R^2^ value of 0.81 aligns with recent machine-learning-enhanced VOC studies (0.80–0.85) that combined sensor arrays with meteorological data [25,27], and surpasses the typical 0.40–0.60 range reported for static PID calibrations [17]. Additionally, the SMAPE of 62.47% and normalized RMSE of 2.07 × 10^−2^ further validate the model’s robustness and stability across diverse emission profiles and meteorological conditions. These metrics provide a more comprehensive evaluation of the model’s performance, addressing the limitations of MAPE in skewed or heteroscedastic data. Importantly, our cost reduction (~90%) exceeds the 50–70% savings reported for simplified GC systems [16], highlighting the economic feasibility of the proposed framework for large-scale industrial deployment.

Five machine learning models were used to evaluate feature importance. The analysis revealed that wind direction and wind speed are the dominant predictors, explaining 38% and 25% of the variance, respectively, in the RF model. The negative correlation between VOC concentration and wind direction (r = −0.17) observed at Site A reflects the combined effects of upwind emission sources and topographic channeling.

During a sudden emission event (>1500 μg m^−3^), the RF model underestimated peak concentrations by 37.8%. This bias is attributed to (i) a scarcity of high-concentration samples in the training set (<0.5%) and (ii) nonlinear PID signal saturation at elevated levels. Similar saturation artifacts have been reported in PID-based soil-flux systems [27]. While the overall MAPE decreased from 129% to 35% after excluding low-concentration samples (<100 μg m^−3^), the high-end bias underscores the need for stratified sampling or transfer learning augmentation in future model updates.

Seasonal factors are likely to have a significant impact on model performance; however, direct comparisons of model performance across different seasons at the same site are not feasible. Variations in temperature and humidity can affect the performance of PID: lower temperatures may cause baseline drift and reduced sensitivity, while higher humidity levels can impair ionization efficiency. These effects tend to be more pronounced during winter and summer. Additionally, seasonal meteorological conditions, such as temperature inversions, can influence pollutant dispersion and the representativeness of monitoring results. Emission control policies implemented in winter may also alter the distribution of VOC concentrations, increasing the proportion of low-concentration samples and thereby affecting the model’s performance and error distribution.

Cross-validation at Sites B and C yielded R^2^ values of 0.68 to 0.69, slightly lower than the 0.81 observed at Site A. This decrease is attributed to lower baseline concentrations (mean 40–90 μg m^−3^ versus 200 μg m^−3^ at Site A) and differing emission profiles (mechanical processing versus pharmaceutical/chemical industries). Nonetheless, RMSE values (28–45 μg m^−3^) remained within acceptable regulatory limits, indicating that the RF model retains practical utility across diverse industrial contexts. Incorporating categorical source-type variables or seasonal calibration cycles could further enhance generalizability.

The developed framework provides a near-real-time cost-effective solution for VOC surveillance in industrial parks. Its minute-level latency enables rapid detection of fugitive emissions, facilitating prompt mitigation measures and supporting dynamic health-risk early-warning systems. A 90% cost reduction allows for the deployment of dense sensor networks, thereby enhancing spatial coverage for source-apportionment studies. Integration of the RF model into lightweight edge devices (Raspberry Pi-class) has been pilot-tested, demonstrating that it maintains prediction accuracy with inference times under 2 s, paving the way for autonomous grid-based monitoring.

## 5. Conclusions

### 5.1. Summary of Conclusions

Compared to the conventional single-coefficient PID calibration method (average R^2^ > 0.40 in our preliminary tests [17]), the RF model demonstrates a 97.6% improvement in prediction accuracy R^2^ = 0.81, a SMAPE = 62.47%, and RMSE_norm_ = 2.07 × 10^−2^, while maintaining comparable hardware costs. These additional metrics provide a more robust evaluation of the model’s performance, particularly in the presence of skewed or heteroscedastic data. This underscores the necessity of multivariate dynamic calibration in industrial applications.

This study developed a low-cost, real-time monitoring system for VOC mass concentration in industrial parks by integrating a PID with an RF algorithm. The main conclusions are as follows:

(1) Validation of the correlation between PID and GC-FID monitoring data: In long-term comparative tests conducted at a typical industrial park (Site A), despite the influence of differences in ionization potentials (IP) of mixed gases on PID signals, the volume fractions (nmol mol^−1^) of VOCs measured using the PID device showed a significant correlation with those measured using the GC-FID system (highest R^2^ = 0.92), indicating that PIDs can effectively capture the dynamic trends of VOC emissions in real-time.

(2) Optimization efficacy and advantage mechanism of the RF algorithm: By integrating multi-dimensional data, such as PID volume fractions, temperature, humidity, and wind speed, the RF model successfully achieved precise conversion from volume fraction (nmol mol^−1^) to mass concentration (μg m^−3^). Its prediction accuracy was significantly superior to that of other algorithms. The core strengths of the RF model lie in its ensemble learning mechanism, which suppresses overfitting in individual trees (with less than a 5% difference in R^2^ between the training and test sets), and its nonlinear modeling capability, which dynamically corrects for meteorological interferences.

(3) Model generalizability and limitations across scenarios: At Sites B and C, which have heterogeneous emission corrects for dominated by residential and light industrial sources, the model continued to demonstrate strong predictive performance, achieving R^2^ values reaching 0.68–0.69. This fully validates the model’s adaptability in the region. However, there are some limitations in high-concentration predictions, especially during sudden pollution events, due to the scarcity of high-value samples in the training dataset.

### 5.2. Future Outlook

This study innovatively developed a rapid-response monitoring system based on a PID sensor to address the technical bottlenecks in online VOC monitoring. Compared to traditional GC-MS methods, the system shows significant improvements in key performance indicators: the real-time response speed has been accelerated to the minute level, which is a 95% reduction from the 30–60 min required by GC-MS. Meanwhile, monitoring costs have been cut to 10% of those of conventional solutions, with equipment purchase costs being below CNY 50,000. However, due to the hardware limitations of the current equipment, we opted for the relatively better-performing Random Forest algorithm. In some cases, other algorithms might perform better. In future research, we will further explore the potential of other algorithms and combine them with the hardware capabilities of new equipment. For example, combining Random Forest with LSTM networks could offer greater advantages. In addition, optimizing hyperparameters and feature selection can also enhance model performance. To address these technical shortcomings and further improve the model’s generalizability, some specific improvements include the following:

Incorporation of SMAPE and RMSEnorma into the model evaluation framework to provide more stable insights across diverse datasets.

Development of feature-level data fusion algorithms, including the construction of dynamic fingerprint spectra for typical VOCs in industrial parks (aromatic hydrocarbons, alkanes, and halogenated hydrocarbons) and the establishment of a transfer learning framework with a pre-training and fine-tuning models to adapt to complex on-site conditions.

Integration of high-precision temperature and humidity compensation modules, including the development of transfer function compensation models, research and development of embedded dynamic calibration systems, and design of dual-channel reference measurement structures with adjustable zero-point calibration intervals (5–30 min) to suppress baseline drift.

Early-warning mechanisms, including developing a two-level early-warning mechanism for concentration exceedances, with thresholds set at 500 μg m^−3^ (primary warning) and 800 μg m^3^ (secondary warning). Compared to the 1000 μg m^−3^ alarm threshold of the GC-FID, this system offers early warning tens of minutes in advance.

## Figures and Tables

**Table 1:** Core advantages and hyperparameter settings of machine learning algorithms.

Algorithm	Core Advantage	Hyperparameter Settings (GridSearchCV Optimization)	Optimization Strategy
SVR	Multivariate linear fitting	Kernel function (linear), penalty coefficient (C = 10)	Maintained linear kernel stability with grid-selected optimal penalty coefficient
PR	Nonlinear fitting capability	Degree (degree = 2)	Optimal degree selected through model complexity testing
DT	High model interpretability	Maximum depth (10), minimum samples split (2)	Pre-pruning strategy to balance depth and overfitting risk
GBDT	Residual iterative optimization	Learning rate (0.1), number of trees (100), maximum depth (4)	Early stopping for iteration control
RF	High stability and anti-overfitting	Number of trees (100), maximum depth (10), maximum features (2)	Feature subsampling to enhance diversity Tree depth optimization for feature interaction

**Table 2:** Comparison results of PID and GC-FID systems. Data collection period for all devices: December 2021 to March 2022. Effective data selection criteria: PID and GC-FID timestamps aligned with no missing data.

Device Name	Effective Data	Mean (nmol mol^−1^)	R^2^	Slope	Intercept	RMSE (nmol mol^−1^)	MAE (nmol mol^−1^)
GC-FID	915	23.4 ± 36.4	1.00	/	/	/	
Y-2	858	50.4 ± 34.7	0.76	0.94	−22.9	30.9	27.0
Z-1	915	74.3 ± 5.7	0.92	6.07	−427.8	59.6	50.9
A-1	915	32.5 ± 40.0	0.81	0.82	−3.3	19.4	9.1
A-2	915	28.2 ± 38.5	0.79	0.84	−0.2	18.7	4.8
A-3	915	33.5 ± 40.2	0.85	0.83	−4.5	18.7	10.1
S-1	915	22.5 ± 19.0	0.67	1.58	−12.1	23.3	−0.9
S-2	915	24.4 ± 19.2	0.69	1.58	−15.2	22.9	1.0
S-3	915	20.7 ± 18.8	0.71	1.63	−10.3	23.1	−2.7

**Table 3:** Performance comparison of machine learning models.

Model Name	MSE	RMSE (μg m^−3^)	MAE	R^2^	MAPE (%)	SMAPE (%)	RMSE_norm_
SVR	2570.33	50.70	27.96	0.79	221.43	81.79	4.23 × 10^−2^
PR	3445.14	58.70	29.52	0.72	231.03	106.85	2.29 × 10^−2^
DT	4254.76	65.23	30.48	0.65	220.86	77.52	4.32 × 10^−2^
GBDT	2504.75	50.05	24.40	0.80	235.28	82.25	2.08 × 10^−2^
RF	2326.23	48.23	20.25	0.81	129.95	62.47	2.07 × 10^−2^

**Table 4:** Time series of concentration pollution event. During the pollution event on 4 March 2022, the model underestimated peak concentrations by 37.8% (1223.4 vs. 1967.68 μg m^−3^).

Time	PID (nmol mol^−1^)	Predicted_FID (μg m^−3^)	FID (μg m^−3^)	Main Pollutant (μg m^−3^)	Main Pollutant Type
4 March 2022 20	117.44	447.9	445.95	128.37	Toluene
4 March 2022 21	869.76	1223.4	1967.68	787.30	1,3-Butadiene
4 March 2022 22	91.99	420.1	305.35	65.09	Isoprene
4 March 2022 23	30.28	76.5	119.83	22.23	Xylene
5 March 2022 00	106.31	434.1	462.49	253.18	Isopentane
5 March 2022 01	82.61	319.6	362.61	74.84	Xylene
5 March 2022 02	96.58	459.6	426.27	103.66	Xylene
5 March 2022 03	161.16	708.6	698.46	272.94	Isopentane
5 March 2022 04	191.02	858.8	803.06	386.08	Isopentane
5 March 2022 05	117.00	453.4	494.36	98.79	Isopentane

**Table 5:** Cross-site analysis of VOC concentrations and model performance.

Site Name	Site A		Site B		Site C	
Device Name	GC-FID	A-3 Prediction	GC-FID	A-1 Prediction	GC-FID	A-2 Prediction
Mean (μg m^−3^)	47.9 ± 110.6	38.6 ± 82.6	28.3 ± 31.3	28.2 ± 22.6	22.3 ± 27.6	20.4 ± 17.6
Training Set	914		1364		1253	
Testing Set	914		1364		1253	
R^2^	0.81		0.69		0.68	
RMSE (μg m^−3^)	48.3		17.4		20.4	
MAE (μg m^−3^)	20.5		8.5		10.6	
MeanCV	0.80 ± 0.02		0.67 ± 0.04		0.66 ± 0.05	
Atm	1024.3 ± 5.2		1013.4 ± 4.1		1011.9 ± 4.3	
Temp (°C)	6.9 ± 3.7		20.2 ± 3.9		20.1 ± 4.1	
Hum	74.0 ± 18.1		68.2 ± 14.0		70.8 ± 15.7	
WS	2.2 ± 1.2		0.5 ± 0.5		1.2 ± 0.7	
WD Standard Deviation	1.25		1.8		1.22	

## Data Availability

Data can be accessed under certain conditions. Interested researchers can request access from the authors and provide a detailed description of their research objectives and data usage plans.
